# Impact of Epicatechin Supplementation on Plasma Proteome Profiles in Obese Men and Women—An Exploratory Approach to Sexual Dimorphism

**DOI:** 10.1002/prca.70027

**Published:** 2025-10-17

**Authors:** Celso Pereira Batista Sousa‐Filho, Allanis Valon Ferreira, Leo Kei Iwai, Alison Felipe Alencar Chaves, Talita Souza Siqueira, Victoria Silva, Guilherme Ribeiro Romualdo, Rosemari Otton

**Affiliations:** ^1^ Interdisciplinary Post‐graduate Program in Health Sciences, Cruzeiro Do Sul University Sao Paulo Brazil; ^2^ Laboratory of Applied Toxinology (LETA) and Center of Toxins Immune‐Response and Cell Signaling (CeTICS), Butantan Institute São Paulo Brazil; ^3^ Department of Clinical Medicine Laboratory of Cellular Genetic and Molecular Nephrology, University of São Paulo, School of Medicine São Paulo Brazil; ^4^ São Paulo State University (UNESP), Experimental Research Unit (UNIPEX) Botucatu Medical School Botucatu Sao Paulo Brazil

**Keywords:** biological processes, epicatechin, obesity, polyphenols, proteome

## Abstract

**Purpose:**

Evidence suggests that consuming epicatechin‐rich green tea can increase metabolism in the body, and this metabolic effect might be linked to weight loss in obese subjects. The precise mechanism by which epicatechin influences weight loss is still unclear. Our goal was to identify a specific signature in the plasma proteins of obese individuals, categorized or not by gender (men and women), and to investigate how epicatechin (EC) supplementation affected them. Additionally, we analyzed anthropometric data to assess the potential anti‐obesity effects of EC and to identify any gender‐related differences that may have emerged.

**Methods:**

In our clinical trial, we provided pure EC (90%) at a daily dosage of 200 mg, administered before the main meal, for three months. The participants were obese men and women with a body mass index (BMI) of 30 kg/m^2^ or higher. We conducted measurements of body dimensions and performed biochemical blood tests before and after the supplementation with EC, also analyzing the proteome in the plasma samples.

**Findings:**

EC supplementation did not alter anthropometric parameters in obese subjects, but it did cause significant molecular changes in their plasma proteome, which varied between men and women. Key proteins like RPL30 were consistently regulated, indicating that EC might activate translational remodeling to adapt to metabolic stress in obesity.

**Conclusions:**

Proteomic profiling reveals early biomarkers of therapeutic efficacy, and future research should examine EC's time‐dependent effects on ribosomal biogenesis and metabolic regulation.

## 1 Introduction

Obesity is defined as an excessive accumulation of fat that poses a health risk. The primary cause of obesity is an energy imbalance between calories consumed and calories expended. Globally, there has been an increase in the consumption of energy‐dense foods high in fats and sugars, alongside a decline in physical activity due to the sedentary nature of many jobs, changes in transportation modes, and increasing urbanization. Obesity is typically assessed using the Body Mass Index (BMI), with a BMI over 25 categorized as overweight and over 30 classified as obese. The prevalence of obesity has significantly increased in recent decades. In 2022, approximately 1 in 8 individuals worldwide lived with obesity, with adult obesity rates more than doubling since 1990 and rates among adolescents quadrupling. Specifically, 2.5 billion adults aged 18 and older were overweight, of which 890 million were classified as obese [1]. Moreover, 37 million children under the age of 5 were overweight in 2022. Obesity is a major risk factor for various non‐communicable diseases, such as cardiovascular diseases (CVD), diabetes, musculoskeletal disorders, and certain cancers. In 2019, an estimated 5 million deaths from noncommunicable diseases were associated with a higher‐than‐optimal BMI.

Addressing obesity requires a comprehensive strategy that involves multiple sectors, including health, agriculture, transportation, urban planning, environment, food processing, distribution, marketing, and education. Effective interventions focus on creating supportive environments and communities that make regular physical activity and healthier dietary choices accessible and affordable for everyone. In this context, since the 1980s, interest in flavonoids has surged, reflecting their vital role in promoting health [2]. These compounds, found in a myriad of plants and plant‐based foods, are closely linked to a decreased risk of CVD and obesity [3]. A diet rich in fresh foods—including fruits, vegetables, cereals, beverages, and grains—has enhanced overall health and extended lifespan [4]. Making informed dietary choices paves the way for a healthier future, highlighting the benefits of nature's offerings.

Epicatechin (EC) is a flavan‐3‐ol, a flavonoid found in natural foods such as cocoa, tea, apples, plums, and berries [5]. It has been reported to have various biological functions, including anti‐inflammatory, antioxidant, antimicrobial, cardioprotective, anti‐diabetic, and neuroprotective activities [2, 4, 6]. Some studies have suggested that consuming EC‐rich green tea can increase metabolism, and this effect might be linked to weight loss [7, 8, 9]. However, the precise mechanism by which EC influences weight loss is still unclear, and further research is needed to understand its effects on obese individuals.

Previous preclinical results suggest that EC can directly activate peroxisome proliferator‐activated receptor gamma (PPARγ), potentially functioning as a partial agonist inducing the browning of white adipose tissue (WAT) [10]. Additionally, EC can indirectly activate PPARα in vitro, as evidenced by purified PPAR‐binding experiments [11]. The activation of PPARγ could trigger gene expression reprogramming, leading white adipocytes to adopt a thermogenic phenotype. Moreover, the observed amelioration of hepatic steatosis could potentially be attributed to the indirect activation of PPARα. PPARs are pivotal nuclear receptor proteins functioning as transcription factors regulating the expression of genes involved in cellular differentiation, development, and the metabolism of carbohydrates, lipids, and proteins [12, 13].

It is important to recognize that while mixtures of polyphenols extracted from different plant species have been found to have positive metabolic effects, the processing method and, consequently, the composition of these mixtures are not standardized worldwide. It would be better, for pharmacological purposes, to identify a single flavonoid as a potential therapeutic compound for achieving human health benefits. However, there is very little data on whether there are differences in the metabolic activities of the specific flavonoid components found in available polyphenol extracts. While blood tests have traditionally been used to assess human health, examining the complete set of plasma proteins (the proteome) has been proposed as a potential tool for diagnosing major diseases such as CVD, diabetes, obesity, and various forms of cancer [14, 15]. In the light of this context, our aim was to assess: (A) the anti‐obesogenic effects of EC, unveiling (B) a potential EC‐related protein signature in the plasma of obese individuals, also investigating any gender‐related implications on the outcomes.

## 2 Materials and Methods

### 2.1 Study Population and Participant Recruitment

In this study, 28 obese individuals (15 men and 13 women), aged 25–45 years with a BMI between 30 and 40 kg/m^2^, from São Paulo state, Brazil, were enrolled. The sample size was based on a study by [16]. The recruitment process included pre‐screening via an online form to collect basic data for BMI calculation. Those who met the inclusion criteria were scheduled for an in‐person visit for medical history, anthropometric measurements, and collection of blood samples. Participants were instructed on the protocol and how and when to take the supplement over three months. All participants provided their informed consent following the Declaration of Helsinki by the World Medical Association (WMA). The study was approved by the Ethics Committee of Cruzeiro do Sul University (protocol number 51461221.1.0000.8084).

### 2.2 Exclusion Criteria and Intervention

The following exclusion criteria were applied: individuals taking medications or having more than three chronic conditions, including diabetes, hypertension, cardiovascular diseases, or any other pathology that could affect the study parameters were excluded. Before the study, all participants underwent a medical history interview to assess alcohol, tobacco, and illicit drug use. Subjects were not under medications or were taking any drugs during the clinical trial. Participants were also instructed to refrain from additional physical exercise during the experimental period and to maintain their usual dietary habits. Individuals who answered “yes” to these criteria were excluded from the study.

After the initial selection, the participants were enrolled in a long‐term study. They received a 90‐day supplementation of 200 mg per day of 90% pure EC (PHD Import, São Paulo, Brazil), provided in capsule form once daily before the main meal. Throughout the supplementation period, participants received medical monitoring to address any side effects or adverse reactions.

### 2.3 Sample Collection and Processing

Blood samples were collected from 28 patients at two different time points: before (T1) and after (T2) the EC supplementation. According to the experimental design, anthropometric data were obtained at these two points. Due to logistical constraints, the collections were not conducted simultaneously. Each participant under fasting conditions in the morning provided five 3 mL EDTA blood tubes. The samples were processed within 3 h of collection. After centrifugation, plasma aliquots were then stored at −80°C for further analysis.


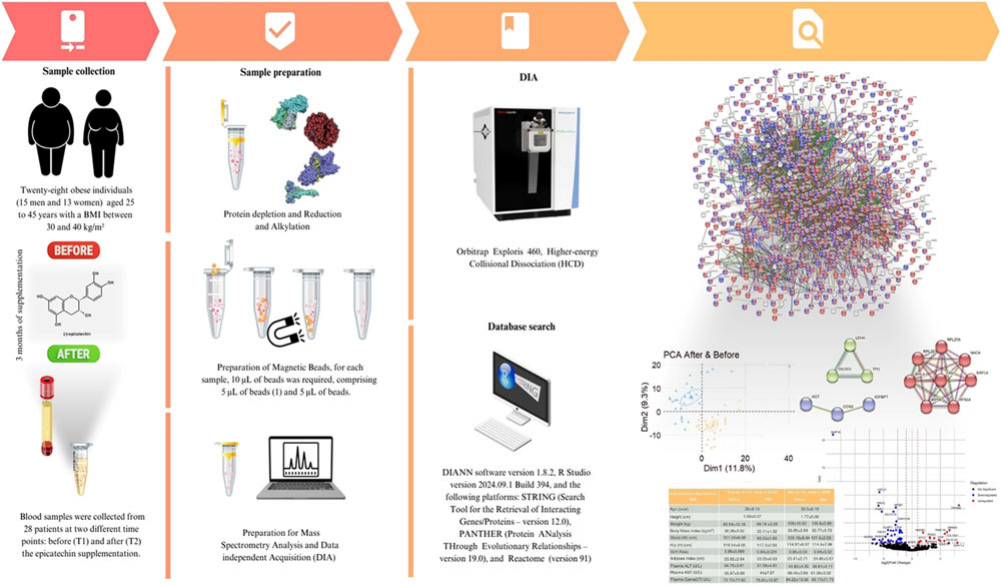


**Experimental Study Design**

### 2.4 Plasma Biochemical Parameter Determination

Plasma samples were used later to determine the levels of glycemia, triglycerides, total cholesterol, GGT (gamma‐glutamyl‐transferase), AST (aspartate aminotransferase), and ALT (Alanine aminotransferase). All procedures followed the manufacturer's instructions (Bioclin, Minas Gerais, Brazil) and samples were read using a multiplate reader Tecan Infinite 200 (Salzburg, Austria).

### 2.5 Plasma Samples Processing for Proteomics

The depletion of the most abundant proteins was based on [16] as outlined in Experimental Design. Plasma samples were prepared by adding 50 µL of plasma followed by 450 µL of ultrapure water and 25 µL of perchloric acid. The mixture was vortexed and centrifuged. The samples were then incubated for 15 min at −20°C, followed by centrifugation at 20,000 × *g* for 1 h at 4°C. The supernatant was carefully recovered, and the pH was adjusted to 8.0 using 2 M HEPES, initially adding 100 µL and adjusting as needed.

The disulfide bonds reduction was performed by adding a fresh dithiothreitol (DTT) solution to achieve a final concentration of 10 mM. The samples were vortexed and incubated at room temperature for 50 min, followed by incubation at 45°C for 10 min in a thermal block. An iodoacetamide (IAA) solution was added to reach a final concentration of 50 mM for protein alkylation. The samples were incubated at room temperature for 1 h, protected from light. To quench the free IAA, a DTT solution was added to a final concentration of 5 mM, and the samples were incubated for 15 min at room temperature and protected from light. The pH was checked to ensure it was between 7.0 and 8.0; if necessary, HEPES was added. The sample cleaning and protein digestion were performed using the Single‐Pot, Solid‐Phase‐enhanced Sample‐Preparation (SP3) protocol [17].

### 2.6 SP3 Protocol

For each sample, paramagnetic beads were prepared [18] to achieve a final volume of 10 µL of bead solution per sample. Following, 50% ethanol was added, and the mixture was incubated in a thermomixer at 24°C for 5 min under agitation at 1000 rpm. The tubes were then fixed in the magnetic rack for 5 min, and the solvent was removed. A total of 180 µL of 80% ethanol was added, and this step was repeated twice for washing. After removing the 80% ethanol, 100 µL of 0.1 M ammonium bicarbonate and 1 µg of trypsin were added. The samples were incubated at 37°C overnight in a thermomixer under agitation at 1000 rpm. Afterward, the tubes were centrifuged at 20,000 × *g* for 1 min at 24°C. They were then fixed in a magnetic rack for 20 min. The solution was carefully removed and transferred to new tubes. The samples were dried in a Speedvac for approximately 2 h and stored at −80°C until further mass spectrometry analysis.

### 2.7 Mass Spectrometry Analysis

The sample mix was resuspended in 20 µL of 0.1% formic acid. Individual samples were resuspended in 10 µL of 0.1% formic acid. The samples were centrifuged at 20,000 × *g* for 5 min at room temperature, and the supernatant was transferred to a new tube, ensuring no beads were present. Corresponding volumes were pipetted into the appropriate plate for the Vanquish Neo coupled to an Orbitrap Exploris 480 system (Thermo). Two microliters of tryptic peptides were loaded onto a trap column (specifications). The data was analyzed in DIA mode with the following parameters: the precursor mass range was between 400 and 800 m/z. Multiplex ion selection was disabled, ensuring individual ion selection within a 10 m/z isolation window, with no overlap between windows. Window placement optimization was turned off, maintaining consistent segmentation across the m/z range, and a total of 40 scan events were configured. The precursors were fragmented with Higher‐energy Collisional Dissociation (HCD) normalized energies set at 32% and 36%. An Orbitrap resolution of 30,000 was used for high mass accuracy, and FAIMS compensation voltage (CV) was set to −45 V. The scan range mode was set to auto, allowing dynamic adjustment, and the RF lens was set at 50% to optimize ion transmission. A custom AGC target was employed, with a normalized AGC target set at 1000%, and a custom maximum injection time of 50 ms was used to balance sensitivity and acquisition speed. Data acquisition was performed with a single microscan per spectrum in centroid mode, and positive ion mode was used throughout. EASY‐IC internal calibration system was enabled to maintain high mass accuracy. Loop control was set to all, ensuring comprehensive coverage of the m/z range within the specified scan events. These settings were optimized to provide high sensitivity, accurate quantitation, and reproducibility across the proteomic samples analyzed.

## 3 Data Analysis

The raw DIA data were converted to mzML using MSConvert (ProteoWizard) with PeakPicking filter activated. The mzML files were processed using DIA‐NN 1.8.2 [19] with a quantification strategy set to “robust LC (high precision)” and with retention time (RT)‐dependent normalization enabled. MS2 and MS1 mass accuracies were set to 10 ppm. The analysis utilized R Studio version 2024.09.1 Build 394 and the following platforms: STRING (Search Tool for the Retrieval of Interacting Genes/Proteins—version 12.0), PANTHER (Protein ANalysis THrough Evolutionary Relationships—version 19.0), and Reactome (version 91). Initially, we conducted a sparsity analysis and performed Little's MCAR test to assess the randomness of the data distribution. After validating the data, we applied a filter to select proteins with at least two valid values per group. Data normalization was carried out using quantile normalization, which involved log base 2 transformations followed by the “normalize.quantiles” method from the R “preprocessCore” package. Data imputation was performed using the BPCA (Bayesian Principal Component Analysis) method with the “pcaMethods” package.

## 4 Statistical Analysis for Proteomics Data

Anthropometric and biochemical results were expressed as mean ± standard error of the mean (SEM). The Shapiro–Wilk and Levene tests were used to verify data normality and variance, respectively, assuming *p* < 0.05. For statistical analysis, a paired *t*‐test was performed comparing after versus before EC. We employed the linear model implemented in the R limma package for statistical analysis for proteome analyses, utilizing empirical Bayesian estimation for variance moderation and correction for false discovery rate (FDR) using the Benjamini–Hochberg method. Student's *t*‐test was performed to evaluate the difference in abundance between groups. A Fisher's exact test was done to evaluate the observation frequency differences. Differential proteins were filtered through fold change (FC) and *t*‐test criteria; differences in protein abundances were considered significant when absolute FC > 1.5 and –log10 of adjusted *p* value < 0.05.

## 5 Results

### 5.1 General Anthropometric and Biochemical Parameters

Anthropometric data from participants in this clinical trial, categorized by gender (women and men), are presented in Table 1. Obese individuals were matched based on age and BMI. After supplementation with EC, no statistical differences were observed between women and men regarding BMI, body weight, waist circumference, hip circumference, and waist‐to‐hip ratio. However, all parameters exhibited a slight decrease following supplementation compared to their values before EC supplementation (see Table 1 and Figure S1). Plasma ALT, AST, and gamma‐GT levels remained unchanged before and after EC supplementation, suggesting no EC‐induced liver implications (Table 1). EC administration did not modify plasma glucose, triglycerides, and total cholesterol (Figure S1) in obese subjects.

**TABLE 1:** Anthropometric features and biochemical data in human plasma before and after EC supplementation.

Anthropometric/biochemical data	Women (*n* = 13, mean ± SEM)		Men (*n* = 15, mean ± SEM)	
	Before	After	Before	After
Age (year)	36 ± 6.15		33.5 ± 6.19	
Height (cm)	1.59 ± 0.07		1.77 ± 0.09	
Weight (kg)	89.54 ± 12.16	89.18 ± 3.28	106 ± 10.52	105.6 ± 2.89
Body mass index (kg/m^2^)	35.26 ± 3.92	35.11 ± 1.02	33.95 ± 2.89	33.77 ± 0.72
Waist (W) (cm)	101.04 ± 8.08	98.25 ± 1.83	109.16 ± 8.94	107.4 ± 2.03
Hip (H) (cm)	118.54 ± 9.05	117.2 ± 2.63	114.97 ± 6.97	114.4 ± 1.96
W/H ratio	0.86 ± 0.086	0.84 ± 0.024	0.95 ± 0.09	0.94 ± 0.02
Adipose index (cm)	23.92 ± 2.64	23.25 ± 0.53	25.41 ± 2.71	24.95 ± 0.67
Plasma ALT (U/L)	34.79 ± 3.61	31.58 ± 4.81	41.62 ± 4.35	39.61 ± 4.11
Plasma AST (U/L)	55.87 ± 9.88	44 ± 7.07	69.45 ± 3.86	61.38 ± 3.02
Plasma GamaGT (U/L)	79.72 ± 11.62	79.81 ± 10.87	94.22 ± 13.56	93.17 ± 11.73

*Note:* cm = centimeter, U/L = Units/Liter.

**FIGURE 1:**
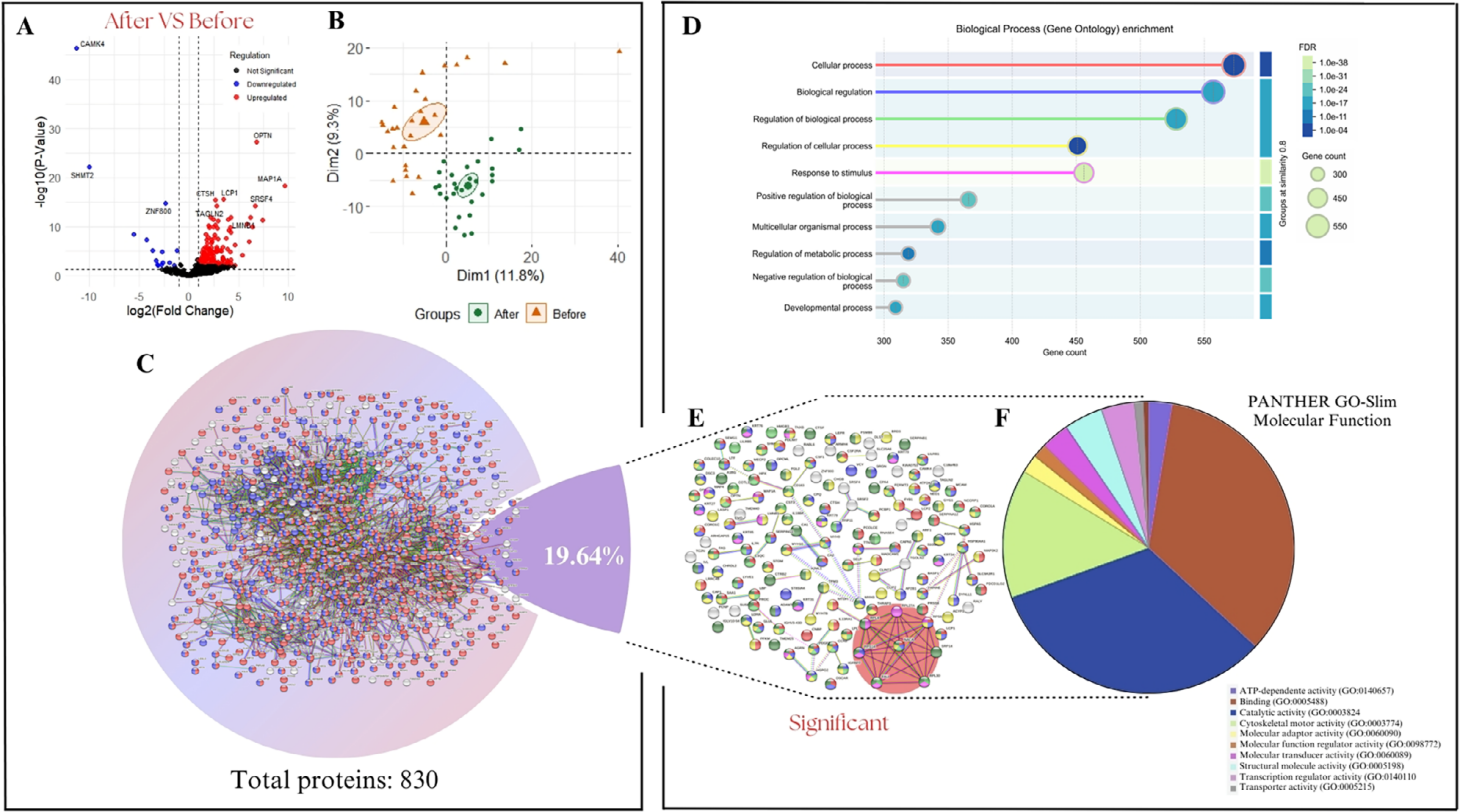
(A) The volcano plot shows the expression of 830 valid proteins in the plasma of obese subjects (*n* = 28) after vs. before EC supplementation, with significant values considered for fold change > 1.5 and –log10 (adjusted *p* value) < 0.05. Red dots indicate upregulated proteins, blue dots indicate downregulated proteins, and black dots indicate proteins with no significant change. (B) Principal component analysis (PCA) of label‐free quantitative proteomics; circles and diamonds indicate, respectively, after and before EC supplementation. (C) Biological pathway enrichment (String platform) analysis conducted on the total proteins from the study (830 proteins), considering a high‐confidence interaction score of 0.700 among all obese participants without gender distinction, comparing after vs. before EC supplementation. (D) Gene Ontology (GO) analyses the top 10 biological processes enriched. Pathways with higher gene counts include cellular process, biological regulation, regulation of biological processes, response to stimuli, positive regulation of biological processes, molecular processes of the organism, metabolic processes, regulation of metabolic processes, and negative regulation of biological processes. (E) Biological pathway enrichment (String platform) analysis conducted with the 163 proteins significantly different in plasma of all obese participants without gender distinction, comparing after vs. before EC supplementation, considering a high‐confidence interaction score of 0.700. Significant values ​​were considered with fold change > 1.5 and with –log10 (adjusted *p* value) < 0.05. (F) Biological pathway enrichment analysis of the 163 proteins significantly modified after EC supplementation loaded in the Panther platform. Nodes represent individual Gene Ontology (GO)—Biological (BP). Significant values ​​were considered with fold change > 1.5 and with –log10 (adjusted *p* value) < 0.05.

### 5.2 Bioinformatics Analysis of Plasma Proteomics in Obese Individuals

To better understand the mechanisms by which EC affects obese individuals, we conducted comprehensive proteome analyses before and after supplementation in obese men and women. This exploratory investigation focuses on two key aspects: (1) evaluating the overall effect of EC treatment without considering gender differences and (2) examining the treatment's impact while specifically addressing gender differences.

### 5.3 Effect of EC Treatment Regardless of Gender

In our first approach, regardless of gender, 895 proteins were identified in plasma samples before normalization and data imputation using a filter requiring two valid values per group (before and after supplementation with EC). After normalization and data imputation, 830 proteins, regardless of gender, were included in the statistical analyses and considered the obese human proteome in plasma (refer to Table S1). The outcomes from this initial exploratory analysis revealed that EC treatment modified 163 proteins (refer to Table S2) in the plasma of obese individuals, with 146 proteins significantly upregulated and 17 proteins downregulated (Figure 1A). Principal Component Analysis (PCA) showed a proteomic profile divergence between individuals after versus before EC supplementation (Figure 1B). The component variations of only 9% to ∼12% can be attributed to an expected diversity of the study's participants, as they are not distinguished by gender.

Enrichment analysis using the STRING platform has uncovered significant interactions among proteins, using a robust interaction score of 0.700 (Figure 1C). Out of the 830 initial proteins loaded into the STRING platform, 73 were not found, leaving us with 757 proteins that exhibited protein‐protein interactions (PPI) (*p* < 1.0e‐16). Next, we conducted an enrichment analysis to identify functional enrichments within the network of the 163 significantly different proteins in plasma. This analysis was done with 163 significantly different proteins in obese subjects following EC supplementation, of which 150 were found in the STRING platform, and 13 were not found (Figure 1E) and PPI enrichment *p* < 0.00017. The Biological Process (BP) Enrichment analysis in Gene Ontology (GO) revealed, biological regulation, regulation of biological processes, response to stimuli, positive regulation of biological processes, multicellular organismal processes, regulation of metabolic processes, negative regulation of biological processes, and developmental processes as top 10 enriched terms in the network (Figure 1D). Figure 1E illustrates the GO slim molecular function pathway of 163 proteins significantly modified and loaded in the Panther platform, indicating that a major proportion of these plasma proteins are involved in binding and catalytic activities.

**FIGURE 2:**
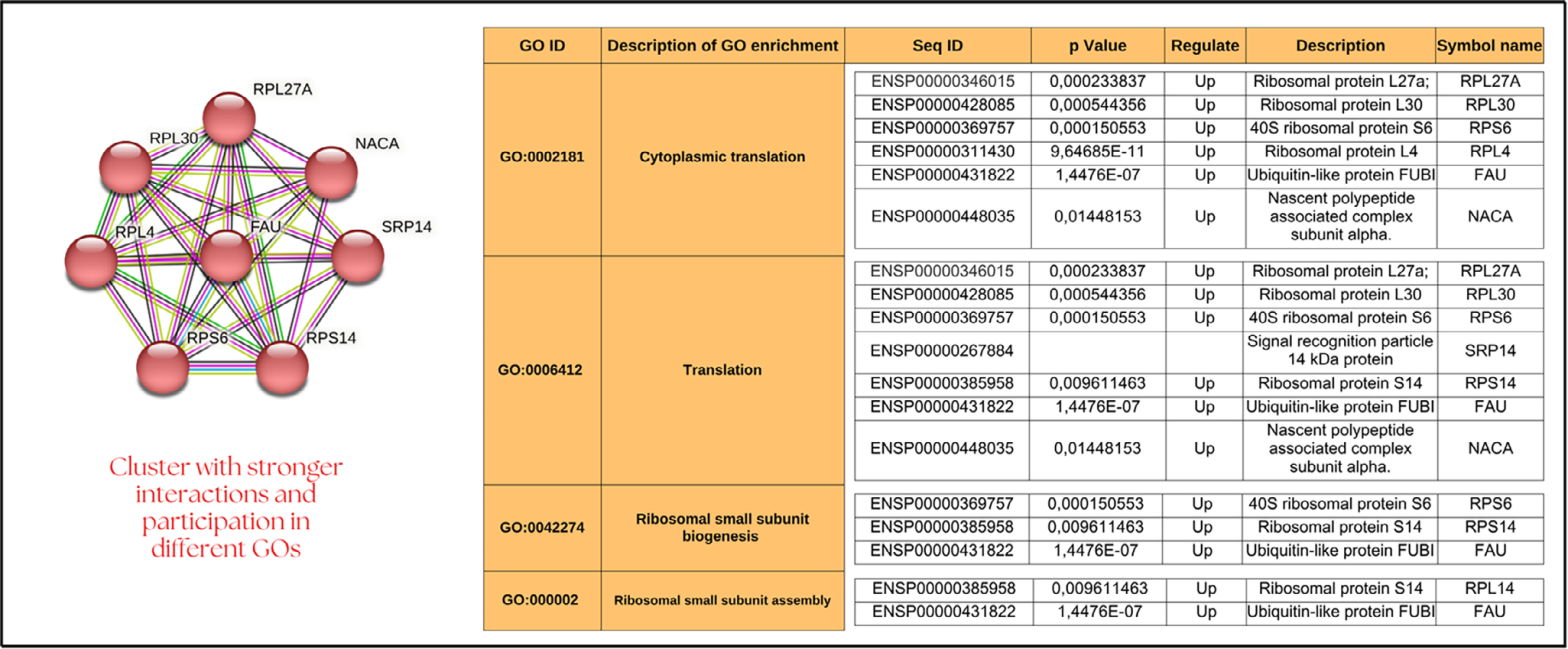
Summary of the main outcomes found after EC supplementation in obese subjects without considering gender. The representative enrichment pathway of eight proteins significantly modified after EC supplementation involved in cytoplasmic translation, translation, ribosomal small subunit biogenesis, and ribosomal small subunit assembly. For more information, please refer to the text. High‐evidence interaction score of 0.700.

Figure 2 summarizes the key findings from the analyses performed on obese subjects, regardless of gender, after EC supplementation. The analysis in the String platform identified key biological processes associated with a cluster of eight proteins involved in ribosomal protein synthesis as followed: Signal Recognition Particle 14 kDa protein (SRP14), Ribosomal Protein L4 (RPL4), Ribosomal Protein L27a (RPL27A), 40S Ribosomal Protein S6 (RPS6), Ribosomal Protein S14 (RPS14), Ribosomal Protein L30 (RPL30), Ubiquitin‐like Protein FUBI (FAU), and Nascent Polypeptide Associated Complex Subunit Alpha (NACA). These proteins are strongly linked in GO biological processes to cytoplasmic translation, translation, ribosomal small subunit biogenesis, and ribosomal small subunit assembly (Figure 2A). All proteins in the cluster were found to be upregulated and achieved a robust interaction score of 0.700, highlighting the potential of EC to modulate the human plasma proteins involved in ribosomal biogenesis. The implications of these outcomes in obese individuals still need to be explored.

While EC supplementation did not induce significant changes in anthropometric parameters or traditional plasma biomarkers, proteomic profiling revealed substantial molecular remodeling, with 163 proteins displaying significant alterations. These findings suggest that, although no overt anti‐obesity effects were observed in three months, EC exerts pronounced molecular changes that may precede systemic metabolic adaptations.

### 5.4 Effect of Epicatechin Treatment: Gender‐Dependent Outcomes

**FIGURE 3:**
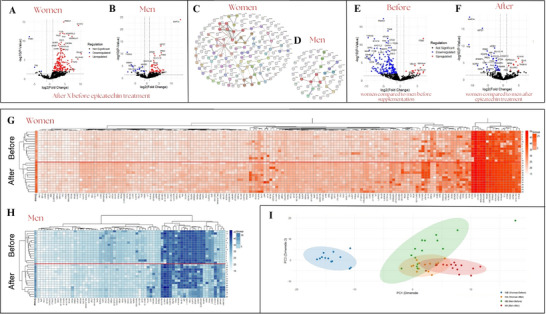
Volcano plot of differentially expressed proteins in plasma of obese women (A) and men (B) after EC treatment. (C) Biological pathway enrichment analysis of the 165 proteins significantly modified after EC supplementation in women and 46 in men (D). Volcano plot of differentially expressed proteins in plasma of women compared with men before epicatechin treatment (E), and women compared with men after epicatechin treatment (F). Red dots represent upregulated proteins, blue dots represent downregulated proteins, and black dots represent proteins with no significant change. (G) The heat map shows that 165 proteins were differentially expressed in women before and after EC, and 67 proteins were expressed in men before and after EC (H). (I) Principal component analysis (PCA) of label‐free quantitative proteomics; circles in different colors indicate, respectively, WB (women before), WA (women after), MB (men before), and MA (men after) EC supplementation. Significant values ​​were considered with fold change > 1.5 and with –log10 (adjusted *p* value) < 0.05. High‐confidence interaction score of 0.700.

In the gender‐based analysis, 768 proteins (Table S3) were identified as valid, meeting at least two valid values per group (men, women, before, and after supplementation). The results showed that women displayed significant differences in 165 (Table S4) proteins after supplementation compared to before, 162 were upregulated, and 3 were downregulated (Figure 3A). In contrast, men showed 67 proteins (Table S5) with significant differences after compared with before EC supplementation, 17 down‐ and 50 upregulated (Figure 3B). By loading significant proteins onto the STRING platform, 165 proteins from women (Figure 3C) and 67 proteins from men (Figure 3D) presenting an interaction score set at 0.700, we found three clusters of proteins in women that were associated with cytoplasmic ribosomal proteins, glyceraldehyde‐3‐phosphate metabolism, and protein synthesis and biogenesis. In contrast, no significant interactions between proteins were identified in men, highlighting a notable sexual dimorphism. When comparing women versus men before EC supplementation, 176 proteins were downregulated in women while 14 were upregulated, resulting in a total of 190 proteins differentially abundant (Figure 3E). Comparing the effects of EC between women and men, we identified 46 proteins in the plasma proteome that were significantly regulated in abundance. Among these, 10 proteins were upregulated, and 36 were downregulated (see Figure 3F).

Figure 3G,H show a heat map illustrating the expression of 165 differentially expressed proteins in women and 46 proteins in men, both before and after EC supplementation. This data highlights significant differences in the proteomes between the two sexes. In women, there was a notable change in protein expression patterns after supplementation, with nearly all proteins being downregulated. In contrast, men had a different response, as many proteins were upregulated following EC supplementation.

Principal component analyses of the data (Figure 3I) revealed that the protein profiles in women before EC supplementation were significantly different from those observed after supplementation. Additionally, these profiles differed from those in men both before and after EC supplementation. In contrast, the plasma protein profiles in men exhibited minimal changes following EC supplementation. Notably, after supplementation, the protein profile in women became very similar to that of men, suggesting that EC may reduce sexual dimorphism in plasma proteins. This outcome indicates a gender‐specific response to epicatechin treatment in obese subjects.

**FIGURE 4:**
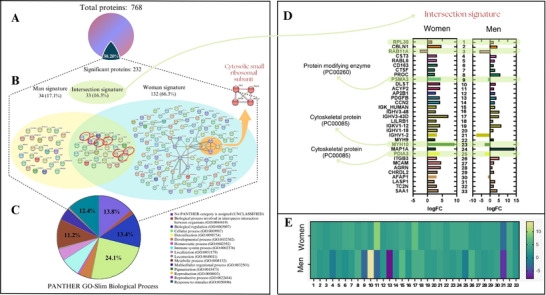
(A) Representative image showing 768 proteins expressed in the plasma of men (*n* = 15) and women (*n* = 13), highlighting 232 proteins (30.2%) that were significantly different in the proteome with a fold change > 1.5 and adjusted *p* value < 0.05. (B) Venn diagram indicating 33 proteins with a common signature expression between women and men after EC supplementation. (C) The 232 proteins loaded onto the Panther platform with GO‐slim Biological Process, highlighting the biological regulation, cellular processes, and metabolic process among the enriched pathways representing the main proteins. (D) The intersection signature of 33 shared proteins between women and men, indicating those with opposite expression between genders, and the main biological process (Protein Class—PC 00260, PC00085, PC 00263) highlighted following the Panther platform. (E) Heatmap with logFC values from the 33 shared proteins in women and men.

From the 768 proteins validated in both women and men, before and after EC supplementation, we created a Venn diagram showing the 67 proteins that were identified in men and 165 in women, totaling 232 proteins (30.2%) with a significant p‐value of less than 0.05 depicted in the String platform (Figure 4A,B). In women, the highlighted proteins exclusive to the female gender belong to a cluster associated with cytosolic small ribosomal proteins. However, in men, no significant interactions were observed among the proteins when considering an interaction score of 0.700. The top Gene Ontology (GO) annotations for the 232 identified proteins, obtained using the Panther platform based on biological processes, and are presented in Figure 4C. These annotations highlight the enriched pathways of modified proteins involved in various biological processes, including biological regulation (13.4%), cellular process (24.1%), metabolic processes (11.2%), and response to stimulus (12.4%) while 13.8% of proteins were not classified in the GO Panther Platform.

Only 33 proteins (16.3%) were found in the intersection shared by both women and men after EC supplementation. The intersection signature of proteins between men and women (Figure 4D,E) revealed that most proteins were upregulated in both genders, as indicated by the log of fold change (logFC). However, four proteins—PMSA3, MYH10, PDIA3, and AFAP1—showed an opposite expression pattern between men and women. Additionally, the RPL30 protein, which is associated with ribosome biosynthesis, was found to be elevated in both men and women. In contrast, the ras‐related protein Rab‐11A (RAB11A) decreased in both genders following EC supplementation. Further investigation is needed to determine whether the shared proteins could serve as biomarkers for EC in plasma.

## 6 Discussion

### 6.1 Proteomic Remodeling in Plasma After EC Supplementation in Obese Subjects

This study advances our understanding of the impact of epicatechin supplementation on the plasma proteome of obese subjects, providing insights into molecular adaptations that may precede overt systemic changes. Rather than beginning by emphasizing the limitations of previous research, our work highlights how exploring the proteomic landscape in response to EC can bridge the gap between preclinical findings and clinical outcomes [20, 21]. In our clinical trial, obese men and women received 200 mg of 90% pure epicatechin daily for 90 days (12 weeks). Although anthropometric and standard biochemical markers remained largely unchanged, plasma proteomic analysis revealed significant molecular alterations—with 163 out of 830 proteins showing differential expression independent of gender—pointing to an early cellular response to EC supplementation.

Importantly, the absence of changes in plasma hepatic enzyme levels (AST, ALT, Gamma‐GT) indicates that these molecular alterations occur without overt liver damage. Nevertheless, specific proteins related to hepatic metabolism were modulated. For instance, a 2.12‐fold increase in dipeptidyl peptidase 4 (DPP4) expression—previously linked to the progression of non‐alcoholic fatty liver disease (NAFLD) [22, 23]—suggests that EC may trigger subtle metabolic shifts. In parallel, a 5.35‐fold rise in serum Amyloid A1 (SAA1) expression underscores a potential inflammatory response involving both liver and adipose tissue. These observations prompt further inquiry into whether such changes represent adaptive mechanisms or early signs of metabolic stress, particularly given concerns over high‐dose flavonoid intake as seen with compounds like EGCG [24].

Beyond hepatic indicators, EC supplementation also influenced proteins involved in cellular stress responses. Notably, the significant upregulation of heat shock protein A5 (HSPA5, logFC 2.61) suggests that EC may enhance mechanisms for maintaining endoplasmic reticulum (ER) homeostasis, serving as a compensatory adaptation to increased metabolic demands [23].

A striking aspect of our findings is the coordinated upregulation of a cluster of ribosomal proteins—including NACA, FAU, SRP14, RPL27A, RPS14, RPL4, RPL30, and RPS6—as depicted in Figure 2. This cluster appears to reflect an adaptive enhancement in ribosomal biogenesis and protein synthesis. For example, NACA (upregulated by 2.76‐fold) is known to modulate transcription and protein synthesis, playing a role in osteoblastic differentiation and stress management [25]. Similarly, the increased expression of FAU (logFC 2.60) not only underscores its canonical role in ribosome assembly but also hints at extra‐ribosomal functions in mitigating oxidative stress [26, 27, 28]. Moreover, the upregulation of SRP14 (logFC 2.92) may optimize protein targeting to the ER, while increases in RPL27A (logFC 1.33) and RPS14 (logFC 1.22) further indicate an enhanced translational capacity and stress adaptation induced by EC. The concurrent rise in RPL4, alongside a notable increase in ATF4 (logFC 4.25), reinforces the concept of EC inducing a coordinated modulation of the protein synthesis machinery—an effect also observed with other polyphenols [29, 30]. Our experiments with obese men and women lead us to a compelling conclusion. While EC may not alter anthropometric measurements, it exerts a profound influence on a critical plasma protein fraction. This modulation will likely trigger significant physiological changes over the long‐term for these individuals, underscoring the importance of EC in their health outcomes.

### 6.2 Sex‐specific Adaptations in Plasma Proteome After EC

Sex‐specific adaptations were evident as well. In women, exclusive modulation of proteins such as NACA (logFC 5.87), FAU (logFC 4.70), RPS14 (logFC 2.75), and RPS6 (logFC 4.29) points to targeted regulation of the cytosolic ribosomal subunits. Given that RPS6 is a known substrate of mTORC1 [31] and is influenced by estrogen [32, 33], these findings suggest an amplified translational response in women. Furthermore, the increased expression of proteins involved in cytoskeletal reorganization and intracellular trafficking—such as RAB11A, Actin filament‐associated protein 1‐like 1 (AFAP1, logFC 2.41), and Myosin‐10 (MYH10, logFC 9.26)—along with elevated PDIA3 (logFC 3.20), indicates a robust cellular adaptation unique to women sex. In contrast, men exhibited a decrease in PDIA3 (logFC ‐3.14) and increase in MAP1A (logFC 13.88) and Complement C1q subcomponent A (C1QA, logFC 5.33), highlighting distinct adaptive responses between sexes [34, 35, 36].

The differential expression of ribosomal proteins RPL30 and RAB11A following epicatechin supplementation holds significant physiological implications, particularly in obesity and metabolic regulation. RPL30, a component of the large ribosomal subunit, is involved in protein synthesis and cellular growth [37]. Its increased expression in both men and women could suggest a heightened ribosomal activity, which may be linked to improved protein synthesis or cellular proliferation in response to epicatechin. This could be particularly relevant in obesity, where dysregulated protein synthesis and cellular growth contribute to inflammation and metabolic dysfunction. RPL30 upregulation might also indicate a shift toward more efficient cellular responses, potentially aiding in improving metabolic processes that are often impaired in obese individuals.

On the other hand, RAB11A, which has been associated with cellular stress responses and the regulation of inflammatory pathways, showed a decreased expression in both sexes. This downregulation could be indicative of a reduction in cellular stress or inflammation, which are key contributors to the pathophysiology of obesity. In obesity, chronic low‐grade inflammation is a hallmark of the condition and plays a central role in the development of insulin resistance and other metabolic disturbances [34]. The reduction in RAB11A could suggest a potential anti‐inflammatory effect of epicatechin supplementation, which may help attenuate the inflammatory environment often seen in obesity, thereby improving metabolic outcomes.

### 6.3 Molecular vs. Systemic Effects of EC over Time

A central question arising from our findings is whether the duration of EC supplementation was sufficient to induce metabolic changes at the molecular level while remaining inadequate to produce noticeable alterations in body composition and systemic plasma markers. The modulation of proteins critical for ribosomal function suggests an early cellular response that could eventually lead to functional metabolic adaptations; however, the precise physiological significance of these molecular changes remains to be fully elucidated.

Another important question is whether the observed proteomic alterations are a direct consequence of EC supplementation or simply a reflection of obesity. A study comparing the serum proteomes of non‐diabetic overweight and obese women with those of age‐matched men found no significant differences in proteins related to ribosomal function or cytoskeletal structure, except for RPS6 [36]. The ribosomal protein changes observed are likely due to EC intervention rather than obesity alone. These results underscore the importance of considering supplementation protocols when comparing studies. Although both 90 days and 12 weeks represent equivalent durations, variations in dosing strategies and compound types can lead to markedly different outcomes. For example, one study administered 900 mg of citrus flavonoids daily to overweight and obese individuals for 12 weeks, resulting in significant reductions in BMI and body fat mass [38]. In contrast, a 10‐day intervention with 165 mg of polyphenols from black tea in healthy adults increased fecal lipid excretion but did not affect anthropometric parameters [39]. These differences suggest that high doses administered over short periods may trigger acute metabolic responses but also carry a risk of tissue injury. In contrast, moderate dosing over an extended period might allow for gradual accumulation in target tissues, leading to more stable metabolic adaptations.

This concept holds clinical significance, especially when considering the pharmacokinetics of phenolic compounds like epicatechin. The rapid absorption and elimination of these compounds necessitate prolonged exposure for the manifestation of meaningful biological effects. For example, studies utilizing [2‐^14^C]‐(–)‐epicatechin have demonstrated its primary absorption in the small intestine, extensive biotransformation into 12 related metabolites (SREMs), peak plasma concentrations (*C*_max_) occurring at sub‐micromolar levels approximately 1 h after ingestion, with subsequent excretion in urine over a 24‐hour period—accounting for around 20% of the ingested dose [40]. A notable limitation in our study is the lack of direct measurements of plasma epicatechin and its metabolites, which would have enriched our understanding of its bioavailability and systemic distribution. While we have conducted a detailed mapping of ribosomal protein expression changes, there is no clear clinical endpoint identified, whether in the form of a validated biomarker or a comprehensive understanding of the underlying molecular mechanisms. However, given that the primary aim of a Phase I study is to establish the safety of an intervention, we believe this has been demonstrated, particularly by the absence of hepatic damage.

### 6.4 Final Considerations

In summary, although EC supplementation did not contribute to reducing anthropometric parameters in obese subjects, it induces significant molecular alterations in the plasma proteome of obese individuals, with notable differences between men and women. The consistent regulation of key proteins such as RPL30—a central component of the ribosome involved in protein synthesis—suggests that EC may activate fundamental mechanisms of translational remodeling, potentially serving as an adaptive response to metabolic stress in obesity. These insights underscore the potential of proteomic profiling to reveal early biomarkers of therapeutic efficacy and highlight avenues for future research exploring the time‐dependent effects of EC and its impact on ribosomal biogenesis and metabolic regulation.

## Author Contributions

Celso Pereira Batista Sousa‐Filho, Victória Silva, Allanis Valon, and Rosemari Otton contributed to the conception and design of the study. Celso Pereira Batista Sousa‐Filho, Victória Silva, Leo Kei Iwai, and Alison Felipe Alencar Chaves performed material preparation, data collection, and analysis. Talita Sousa Siqueira performed bioinformatic analyses. The first draft of the manuscript was written by Rosemari Otton and Allanis Valon and revised by Guilherme Ribeiro Romualdo. All authors commented on previous versions of the manuscript. All authors read and approved the final version of the manuscript.

## Conflicts of Interest

All authors of the present manuscript declare that there is no actual or potential conflict of interest, including any financial, personal, or other relationships with other people or organizations that could inappropriately influence or be perceived to influence our work.

## Supporting information




**Supporting Figure 1:** prca70027‐sup‐0001‐FigureS1.docx.

**Supporting Table 1:** prca70027‐sup‐0002‐Tables.xlsx.

## Data Availability

The mass spectrometry proteomics data have been deposited to the ProteomeXchange Consortium via the PRIDE partner repository with the dataset identifier PXD062541 and 10.6019/PXD062541.

## References

[1] World Health Organization . “One in Eight People Are Now Living With Obesity [Internet].” World Health Organization; 2024 Mar 1 [cited 2024 Mar 31], https://www.who.int/news/item/01‐03‐2024‐one‐in‐eight‐people‐are‐now‐living‐with‐obesity.

[2] J. M. Harnly , R. F. Doherty , G. R. Beecher , et al., “Flavonoid Content of U.S. Fruits, Vegetables, and Nuts,” Journal of Agricultural and Food Chemistry 54 (2006): 9966–9977, 10.1021/jf061478a. 17177529

[3] J. I. Dower , J. M. Geleijnse , P. C. H. Hollman , S. S. Soedamah‐Muthu , and D. Kromhout , “Dietary Epicatechin Intake and 25‐y Risk of Cardiovascular Mortality: The Zutphen Elderly Study,” American Journal of Clinical Nutrition 104 (2016): 58–64, 10.3945/ajcn.115.128819. 27225434

[4] A. T. Khalaf , Y. Wei , S. J. A. Alneamah , et al., “What Is New in the Preventive and Therapeutic Role of Dairy Products as Nutraceuticals and Functional Foods?,” BioMed Research International 2021 (2021): 1–9, 10.1155/2021/8823222. 33681381 PMC7925044

[5] M. Prakash and B. V. Basavaraj , “Biological Functions of Epicatechin: Plant Cell to Human Cell Health,” Journal of Functional Foods 52 (2019): 14–24, 10.1016/j.jff.2018.10.021.

[6] M. K. Mandal and A. J. Domb , “Antimicrobial Activities of Natural Bioactive Polyphenols,” Pharmaceutics 16 (2024): 718, 10.3390/pharmaceutics16060718. 38931842 PMC11206801

[7] S. G. West , M. D. McIntyre , M. J. Piotrowski , et al., “Effects of Dark Chocolate and Cocoa Consumption on Endothelial Function and Arterial Stiffness in Overweight Adults,” British Journal of Nutrition 111 (2014): 653–661, 10.1017/S0007114513002912. 24274771

[8] T. Sano , S. Nagayasu , S. Suzuki , et al., “Epicatechin Downregulates Adipose Tissue CCL19 Expression and Thereby Ameliorates Diet‐Induced Obesity and Insulin Resistance,” Nutrition Metabolism and Cardiovascular Diseases 27 (2017): 249–259, 10.1016/j.numecd.2016.11.008. 28062181

[9] E. Cremonini , Z. Wang , A. Bettaieb , et al., “Epicatechin Protects the Intestinal Barrier From High Fat Diet‐Induced Permeabilization: Implications for Steatosis and Insulin Resistance,” Redox Biology 14 (2018): 588–599, 10.1016/j.redox.2017.11.002. 29154190 PMC5691220

[10] E. Cremonini , C. G. Fraga , and P. I. Oteiza , “(–)‐Epicatechin in the Control of Glucose Homeostasis: Involvement of Redox‐Regulated Mechanisms,” Free Radical Biology and Medicine 130 (2019): 478–488, 10.1016/j.freeradbiomed.2018.11.010. 30447350

[11] A. P. Bolin , C. P. B. Sousa‐Filho , M. P. Marinovic , A. C. Rodrigues , and R. Otton , “Polyphenol‐Rich Green Tea Extract Induces Thermogenesis in Mice by a Mechanism Dependent on Adiponectin Signaling,” Journal of Nutritional Biochemistry 78 (2020): 108322, 10.1016/j.jnutbio.2019.108322. 32120266

[12] M. P. Marinovic , C. Pereira , B. Sousa‐Filho , F. Aparecida , and H. Batista , “Green Tea Extract Increases Adiponectin and PPAR α Levels to Improve Hepatic Steatosis,” Journal of Nutritional Biochemistry 103 (2022): 108957, 10.1016/j.jnutbio.2022.108957. 35134507

[13] L. Michalik , J. Auwerx , J. P. Berger , et al., “International Union of Pharmacology. LXI. Peroxisome Proliferator‐Activated Receptors,” Pharmacological Reviews 58 (2006): 726–741, 10.1124/pr.58.4.5. 17132851

[14] X. Wang , Y. Y. Ouyang , J. Liu , and G. Zhao , “Flavonoid Intake and Risk of CVD: A Systematic Review and Meta‐Analysis of Prospective Cohort Studies,” British Journal of Nutrition 111, no. 1 (August 2013): 1–11. 23953879 10.1017/S000711451300278X

[15] P. E. Geyer , L. M. Holdt , D. Teupser , and M. Mann , “Revisiting Biomarker Discovery by Plasma Proteomics,” Molecular Systems Biology 13, no. 9 (September 2017): 942. 28951502 10.15252/msb.20156297 PMC5615924

[16] C. Zhou , K. L. Simpson , L. J. Lancashire , et al., “Statistical Considerations of Optimal Study Design for human Plasma Proteomics and Biomarker Discovery,” Journal of Proteome Research 11 (2012): 2103–2113, 10.1021/pr200636x. 22338609 PMC3320746

[17] A. Viode , P. van Zalm , K. K. Smolen , et al., “A Simple, Time‐ and Cost‐Effective, High‐Throughput Depletion Strategy for Deep Plasma Proteomics,” Science Advances 9 (2023): 1–6, 10.1126/sciadv.adf9717. PMC10058233 36989362

[18] C. S. Hughes , S. Moggridge , T. Müller , P. H. Sorensen , G. B. Morin , and J. Krijgsveld , “Single‐Pot, Solid‐Phase‐Enhanced Sample Preparation for Proteomics Experiments,” Nature Protocols 14 (2019): 68–85, 10.1038/s41596-018-0082-x. 30464214

[19] V. Demichev , C. B. Messner , S. I. Vernardis , K. S. Lilley , and M. Ralser , “DIA‐NN: Neural Networks and Interference Correction Enable Deep Proteome Coverage in High Throughput,” Nature Methods 17 (2020): 41–44, 10.1038/s41592-019-0638-x. 31768060 PMC6949130

[20] A. Börsig , N. Konar , and S. Dalabasmaz , “A Model Study on the Site‐Specificity of (−)‐Epicatechin‐Induced Reactions in β‐Lactoglobulin by High‐Resolution Mass Spectrometry in Combination With Bioinformatics,” Food Chemistry 408 (May 2023): 135242. 36566544 10.1016/j.foodchem.2022.135242

[21] R. Otton , A. Paola Bolin , L. D. Ferreira , and M. A. Mori , “Polyphenol‐Rich Green Tea Extract Improves Adipose Tissue Metabolism by Down‐Regulating miR‐335 Expression and Mitigating Insulin Resistance and Inflammation,” Journal of Nutritional Biochemistry 57 (July 2018): 170–179. 29734116 10.1016/j.jnutbio.2018.03.024

[22] L. Niu , P. E. Geyer , N. J. Wewer Albrechtsen , L. L. Gluud , A. Santos , and S. Doll , “Plasma Proteome Profiling Discovers Novel Proteins Associated With Non‐Alcoholic Fatty Liver Disease,” Molecular Systems Biology 15 (March 2019): 8793. 10.15252/msb.20188793 PMC6396370 30824564

[23] P. Peerapen , C. Chanthick , and V. Thongboonkerd , “Quantitative Proteomics Reveals Common and Unique Molecular Mechanisms Underlying Beneficial Effects of Caffeine and Trigonelline on Human Hepatocytes,” Biomedicine & Pharmacotherapy 158 (February 2023): 114124. 36521247 10.1016/j.biopha.2022.114124

[24] Z. Mostofinejad , E. Cremonini , J. Kang , and P. I. Oteiza , “Effects of (−)‐Epicatechin on Hepatic Triglyceride Metabolism,” Food & Function 15, no. 1: 326–337, https://pubs.rsc.org/en/content/articlelanding/2024/fo/d3fo03666a. 38086683 10.1039/d3fo03666a

[25] E. Hector and D. Cairns , “Evaluation of NACA and diNACA in Human Cystinosis Fibroblast Cell Cultures as Potential Treatments for Cystinosis,” Orphanet Journal of Rare Diseases 17, no. 1: 231, https://pubmed.ncbi.nlm.nih.gov/35710564/. 10.1186/s13023-022-02367-w PMC9205078 35710564

[26] V. Tomati , E. Pesce , E. Caci , et al., “High‐Throughput Screening Identifies FAU Protein as a Regulator of Mutant Cystic Fibrosis Transmembrane Conductance Regulator Channel,” Journal of Biological Chemistry 293, no. 4 (November 2017): 1203–1217, https://pmc.ncbi.nlm.nih.gov/articles/PMC5787799/. 29158263 10.1074/jbc.M117.816595 PMC5787799

[27] W. W. Sun , X. M. Yan , Q. Shi , et al., “Downregulated RPS‐30 in *Angiostrongylus cantonensis* L5 Plays a Defensive Role Against Damage Due to Oxidative Stress,” Parasites & Vectors 13, no. 1 (September 2020): 617, https://pubmed.ncbi.nlm.nih.gov/33298148/. 33298148 10.1186/s13071-020-04495-3 PMC7724845

[28] A. Thai , C. Doescher , N. Kamal , et al., “Single Cell Transcriptomics Profiling of the Stromal Cells in the Pathologic Association of Ribosomal Proteins in the Ischemic Myocardium and Epicardial Fat,” Cell and Tissue Research 399, no. 2 (February 2025): 173–192, https://pubmed.ncbi.nlm.nih.gov/39641799/. 39641799 10.1007/s00441-024-03933-3 PMC11787193

[29] J. Kang , N. Brajanovski , K. T. Chan , J. Xuan , R. B. Pearson , and E. Sanij , “Ribosomal Proteins and Human Diseases: Molecular Mechanisms and Targeted Therapy,” Signal Transduction and Targeted Therapy [Internet] 6, no. 1 (August 2021): 1–22. 34462428 10.1038/s41392-021-00728-8 PMC8405630

[30] C. Chanthick and V. Thongboonkerd , “Comparative Proteomics Reveals Concordant and Discordant Biochemical Effects of Caffeine Versus Epigallocatechin‐3‐Gallate in Human Endothelial Cells,” Toxicology and Applied Pharmacology 378, no. 378 (September 2019): 114621. 31195006 10.1016/j.taap.2019.114621

[31] X. T. Tan , D. B. Teh , L. C. Lau , et al., “#134: Unraveling the mTORC1 Mediated Pathway for Reproductive Longevity Rejuvenation by Targetting RPS6,” Fertility & Reproduction 05, no. 04 (December 2023): 577–579.

[32] M. Holz , “Crosstalk Between mTOR and Estrogen Signaling,” FASEB Journal 34, no. S1 (April 2020): 1.

[33] L. Jiao , Y. Liu , X. Y. Yu , et al., “Ribosome Biogenesis in Disease: New Players and Therapeutic Targets,” Signal Transduction and Targeted Therapy 8, no. 1 (January 2023): 15. 36617563 10.1038/s41392-022-01285-4 PMC9826790

[34] A. Antiguas and M. Dunnwald , “Rab11A Forms a Complex With Interferon Regulatory Factor 6 in Recycling Endosomes,” The FASEB Journal 36, no. S1 (May 2022), 10.1096/fasebj.2022.36.S1.R4164.

[35] N. D. Schartz and A. J. Tenner , “The Good, the Bad, and the Opportunities of the Complement System in Neurodegenerative Disease,” Journal of Neuroinflammation 17, no. 1 (November 2020), https://jneuroinflammation.biomedcentral.com/articles/10.1186/s12974‐020‐02024‐8. 10.1186/s12974-020-02024-8 PMC7690210 33239010

[36] N. M. Al‐Daghri , O. S. Al‐Attas , H. E. Johnston , et al., “Whole Serum 3D LC‐nESI‐FTMS Quantitative Proteomics Reveals Sexual Dimorphism in the Milieu Intérieur of Overweight and Obese Adults,” Journal of Proteome Research 13, no. 11 (July 2014): 5094–5105. 25072778 10.1021/pr5003406

[37] J. Abelson , “The Discovery of Catalytic RNA,” Nature Reviews Molecular Cell Biology 18, no. 11 (October 2017): 653. 10.1038/nrm.2017.105 29018284

[38] H. Ashigai , Y. Taniguchi , M. Suzuki , et al., “Fecal Lipid Excretion After Consumption of a Black Tea Polyphenol Containing Beverage–Randomized, Placebo‐Controlled,” Double‐Blind 39, no. 5 (January 2016): 699–704. 10.1248/bpb.b15-00662 26887502

[39] S. J. Park , A. Sharma , M. H. Bae , et al., “Efficacy and Safety of Sinetrol‐XPur on Weight and Body Fat Reduction in Overweight or Obese Adults: A 12‐Week, Randomized, Double‐Blind, Parallel, Placebo‐Controlled Trial,” Journal of Medicinal Food 23, no. 3 (March 2020): 335–342. 32130058 10.1089/jmf.2019.4649

[40] G. Borges , J. I. Ottaviani , J. J. J. van der Hooft , H. Schroeter , and A. Crozier , “Absorption, Metabolism, Distribution and Excretion of (−)‐Epicatechin: A Review of Recent Findings,” Molecular Aspects of Medicine 61 (June 2018): 18–30. 29126853 10.1016/j.mam.2017.11.002

