# Supplementary material for: Impact of Epicatechin Supplementation on Plasma Proteome Profiles in Obese Men and Women—An Exploratory Approach to Sexual Dimorphism
Source: Proteomics Clin Appl. 2025 Oct 17;20(1):e70027. doi: 10.1002/prca.70027 (PMC12743591; doi:10.1002/prca.70027)

**Figure 1S:** Effects of epicatechin supplementation on plasma biochemical parameters in obese men and women. The bar graphs display the levels of glucose (mg/dL), triglycerides (mg/dL), total cholesterol (mg/dL), aspartate aminotransferase (AST, U/L), gamma-glutamyl transferase (Gamma GT, U/L), and alanine aminotransferase (ALT, U/L) measured before and after the intervention. The data are presented as the mean ± standard error of the mean (SEM). Analyses were conducted separately for women (purple, n=13), men (blue, n=15), and the total study population (green, n=28). Statistical comparisons were performed using paired t-tests within each group to assess the impact of the supplementation.


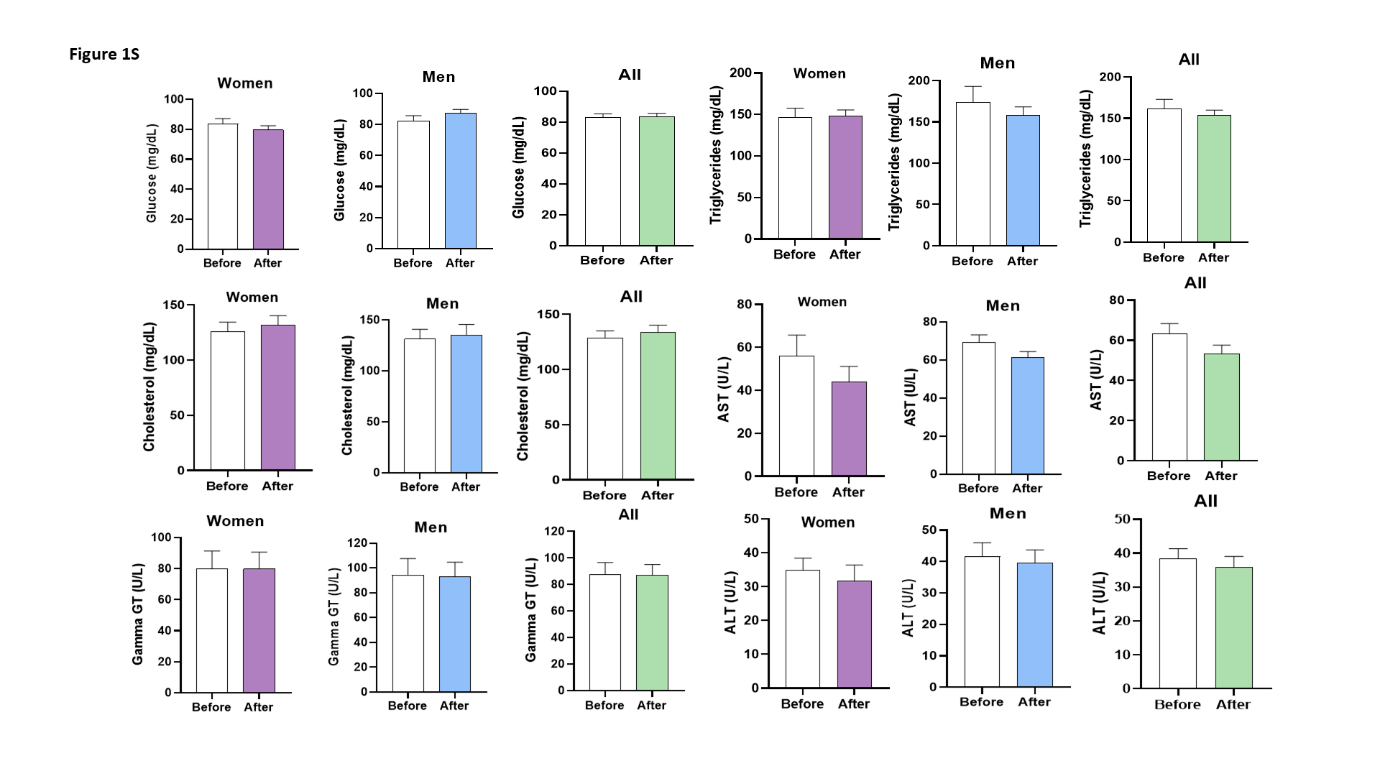

Supplement: Supplementary file 1 — Supporting Figure 1: prca70027‐sup‐0001‐FigureS1.docx. [file PRCA-20-e70027-s002.docx]
